# Supplementary figures and images for: Development of a Wet-Granulated Sourdough Multiple Starter for Direct Use
Source: Foods. 2022 Apr 28;11(9):1278. doi: 10.3390/foods11091278 (PMC9105756; doi:10.3390/foods11091278)

Figure S1

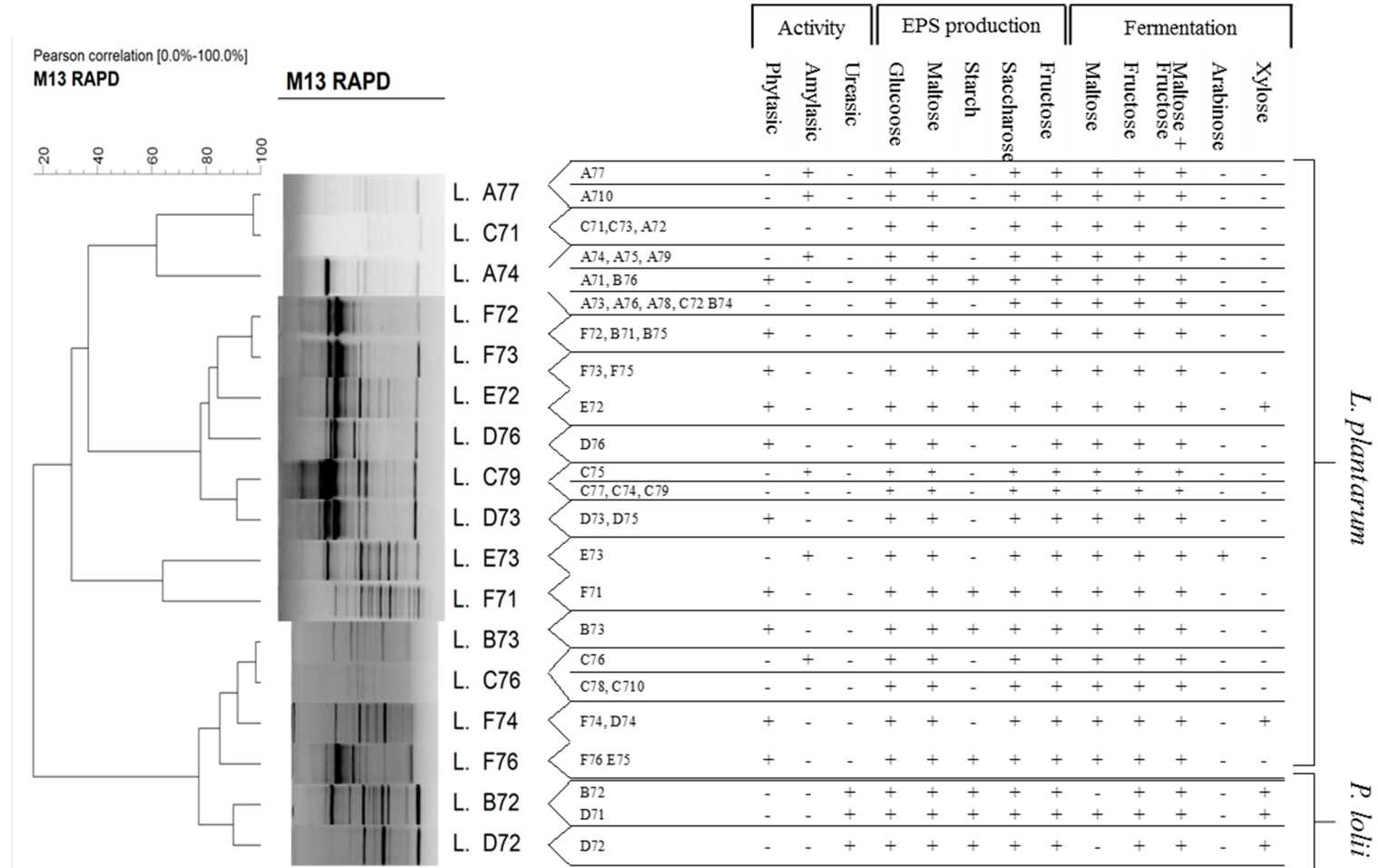

Figure S2

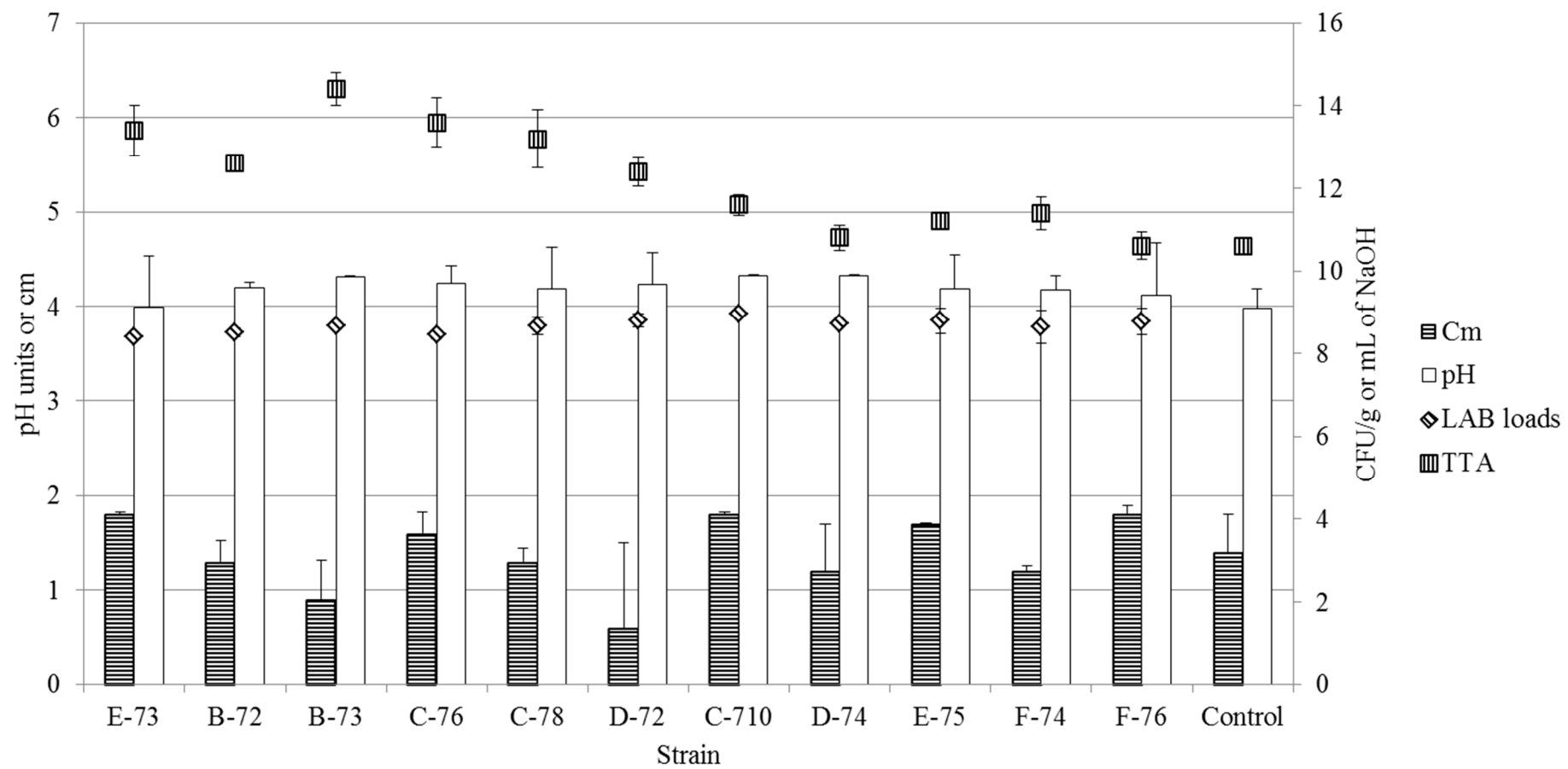

Figure S3

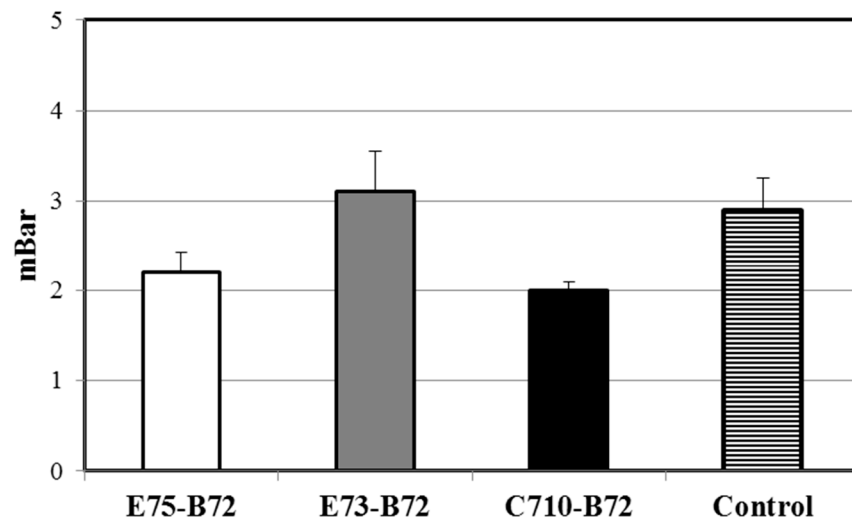

Figure S4

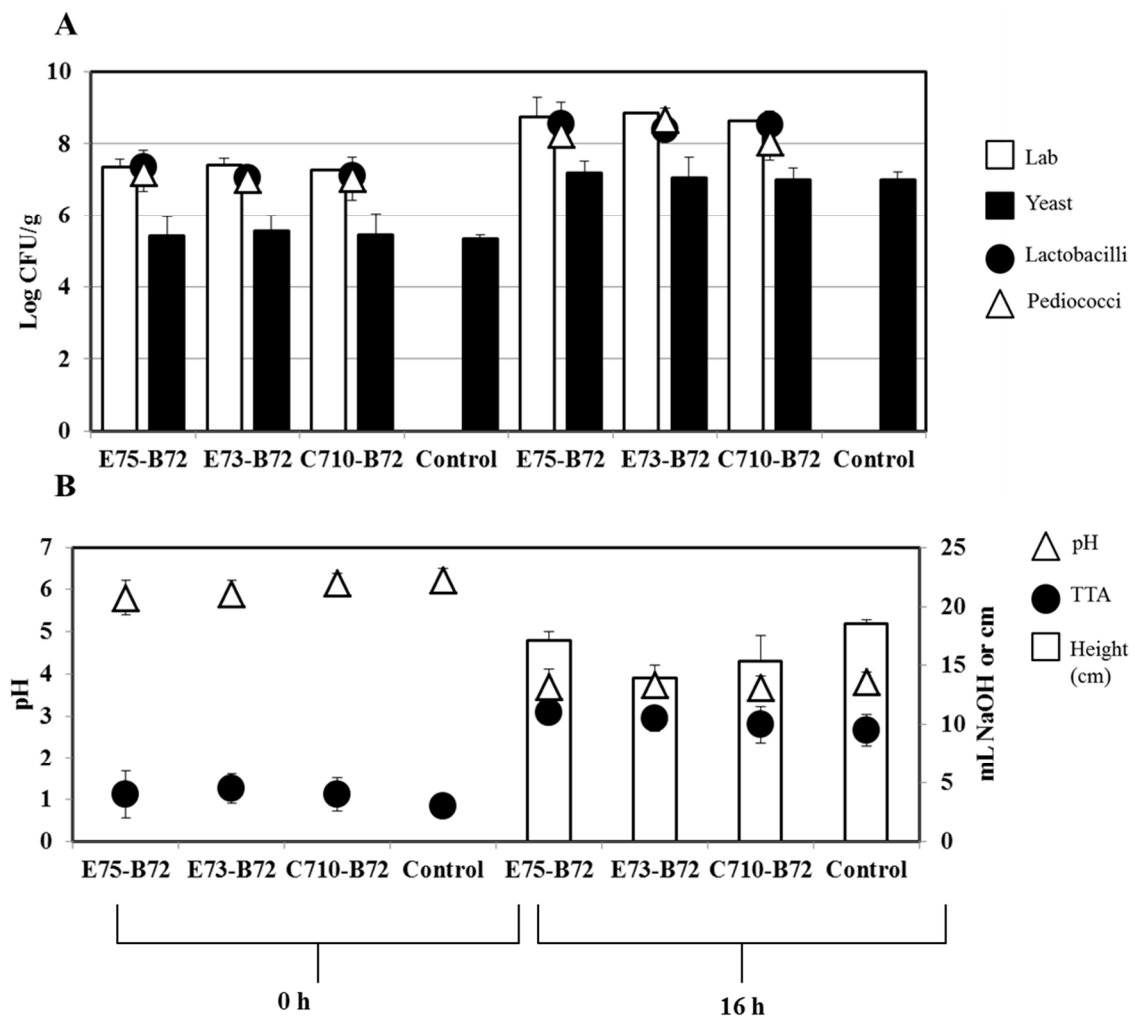

Supplement: Supplementary file 1 [file foods-11-01278-s001.zip › foods-1631034-supplementary.pdf]
